# Dizziness in a tertiary neurological department: A cross‐sectional study

**DOI:** 10.1002/brb3.2864

**Published:** 2022-12-29

**Authors:** Youjin Shen, Wentao Liu, Xiaokun Qi

**Affiliations:** ^1^ The Second School of Clinical Medicine Southern Medical University Guangzhou China; ^2^ Department of Neurology Deqing People's Hospital Zhaoqing China; ^3^ Department of Emergency Huhhot First Hospital Huhhot China; ^4^ Department of Neurology The Sixth Medical Center of PLA of Chinese General Hospital Beijing China

**Keywords:** disequilibrium, dizziness, light‐headedness, stroke, vertigo

## Abstract

**Background and objective:**

Dizziness is a common and challenging symptom, which can be caused by different pathophysiological mechanisms and might affect a large number of population. However, up to now, there have been limited research on the characteristics of dizziness as the chief complaint in hospitalized patients in the Department of Neurology. Thus, the aim of this study was to investigate the hospitalized patients with dizziness as their chief complaint in the Department of Neurology.

**Methods:**

In this cross‐sectional study, we conducted a retrospective document analysis of hospitalized patients admitted to a tertiary neurological department with the symptom of dizziness during the period of September 2019 to December 2020. We included 211 patients with dizziness as their chief complaint from 1841 patients admitted to this tertiary neurological department during that period.

**Results:**

Of all 1841 hospitalized patients, those with dizziness as the chief complaint accounted for 11.5% and most of their past medical history included hypertension, diabetes, cerebrovascular diseases, dyslipidemia, and coronary heart disease. Among these 211 patients, dizziness was more common in women than in men (*p* = .004). More patients presented with vertigo (40.8%) and light‐headedness (39.8%) than disequilibrium (17.1%) and pre‐syncope (2.4%). Nausea (48.3%), vomiting (34.1%), headache (13.3%), walking unsteadily (13.3%), and ear symptoms (12.8%) were the most common concomitant symptoms. Dix‐Hallpike test (24.6%) and Romberg's sign (11.4%) were positive in these dizzy patients. Nystagmus (2.4%), vision changes (1.4%), and hearing disorders (8.5%) were relatively rare symptoms. Common auxiliary examinations were performed, such as magnetic resonance imaging (60.2%), computed tomography (31.8%), carotid duplex ultrasound (30.8%), and echocardiography (28.0%). Benign paroxysmal positional vertigo (24.2%) and stroke/transient ischemic attack (19.0%) were confirmed to be common causes of dizziness. Note that 97.2% of dizzy patients were in improved recovery after treatment.

**Conclusion:**

The diagnosis and management of dizziness remain a challenge for clinicians. Vertigo and light‐headedness were the most common symptoms among different types of dizziness. Benign paroxysmal positional vertigo and stroke/transient ischemic attack were among the leading causes for common dizziness disorders. The prognosis of most dizzy patients was good.

## Graphical abstract

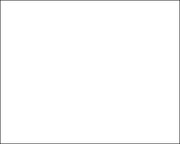

## 1 INTRODUCTION

Dizziness is the third major complaint among individuals reporting health problems following fever and headache (Martins et al., 2017). Dizziness (including vertigo) affects about 15% to over 20% of adults annually in large population‐based studies (Neuhauser, 2016). Dizziness as a common and challenging symptom is widely seen in outpatients, emergency patients, and hospitalized patients. More than one‐third of Americans visit their health physician due to dizziness during their lifetime (Agrawal et al., 2009). Although most of dizziness result from benign causes, some life‐threatening causes also need to be excluded. There are some rare life‐threatening diseases in dizzy patients (with cerebrovascular disease accounting for 6%, cardiac arrythmia for 1.5%, and brain tumor for <1%) (Kroenke et al., 2000; Kwong & Pimlott, 2005). Additionally, dizziness usually leads to some other complications such as falling downs and some other accidents, which might significantly affect the patient's quality of life. However, clinicians are now still unable to correctly diagnose and treat dizzy patients. In addition to Emergency Departments and Otolaryngology Departments, our neurologists often receive and treat patients with dizziness as the chief complaint. However, to date, there have been a handful of investigations of patients with dizziness in neurology. Thus, this study was designed to investigate the hospitalized patients with dizziness in Neurology Department from the aspects of demographic characteristics, characteristics of symptoms, past medical history, physical examination and auxiliary examination, clinical diagnosis, and treatment effect.

## 2 METHODS

In this 16‐month retrospective cross‐sectional study, the documents of all hospitalized dizzy patients admitted to the Department of Neurology of the Sixth Medical Center of Chinese PLA General Hospital, Beijing, China, from September 2019 to December 2020, were evaluated. Of all hospitalized patients with dizziness as the chief complaint admitted to our neurological department during this period of time, we excluded patients who had symptoms of dizziness but did not report it as their chief complaint. Demographic data, baseline characteristics of the patients (different types of dizziness, past medical history, concomitant symptoms), physical examination and auxiliary examination, clinical diagnosis, and treatment effect were recorded using a checklist. Patients were diagnosed based on the findings of symptoms, past history, auxiliary examination, and clinical examination such as presence or absence of headache, tinnitus, hearing loss, nystagmus characteristics, signs of sympathetic release, focal neurologic findings, and so forth. The diagnosis of dizziness conformed to the latest international diagnostic standards. The final decision on the diagnosis of dizziness was made based on the results of brain imaging or para‐clinical findings and the opinion of an expert neurologist. We declared that the study was approved by our hospital ethics committee. The researchers adhered to the principles of Helsinki Declaration and confidentiality of patient information over the course of the study.

### 2.1 Statistical analysis

Data analyses were performed using Statistical Package for the Social Sciences (SPSS) (version 25: SPSS, Inc, an IBM Company, Chicago, Illinois). Normally distributed continuous variables were reported as mean value ± standard deviation; differences were assessed by a two‐tailed *t* test. Categorical variables were introduced as frequency and percentage; a chi square test or Fisher's exact test was performed to establish differences between groups. Significance level was considered *p* < .05.

## 3 RESULTS

### 3.1 Demographic characteristics

During the period of this study, 1841 patients were admitted to our department of neurology with the mean age of 65.92 ± 18.612 years (ranging from 7 to 95) and 54.6% male and 45.4% female. Among them, 211 patients reporting dizziness as their chief complaint were included, with the mean age of 58.76 ± 14.817 years (ranging from 17 to 89) and 44.1% male and 55.9% female. Table 1 shows the number of hospitalized patients with dizziness as the chief complaint in different age groups, indicating that the occurrence of dizziness was significantly higher in women than in men (*p* = .004), while no significant difference in frequency was observed between male cases and female cases in each age group (*p* = .549).

**TABLE 1:** The number of hospitalized patients with dizziness in different age groups (years)

Age	Male (%)	Female (%)	Total (%)
<30	3 (1.4)	6 (2.8)	9 (4.3)
30–39	5 (2.4)	10 (4.7)	15 (7.1)
40–49	14 (6.6)	16 (7.6)	30 (14.2)
50–59	23 (10.9)	27 (12.8)	50 (23.7)
60–69	20 (9.5)	35 (16.6)	55 (26.1)
70–79	22 (10.4)	17 (8.1)	39 (18.5)
≥80	6 (2.8)	7 (3.3)	13 (6.2)

### 3.2 Characteristics of symptoms

The distribution of different types of dizziness was described with more patients presenting with vertigo (40.8%) and light‐headedness (39.8%) than disequilibrium (17.1%) and pre‐syncope (2.4%). More female patients than male patients presented with vertigo (28.0% vs. 12.8%), whereas the ratio of females to males was roughly the same among those with light‐headedness (21.3% vs. 18.5%) and pre‐syncope (1.4% vs. 0.9%). A significant difference was observed between males and females (*p* = .001). Nausea (48.3%), vomiting (34.1%), headache (13.3%), walking unsteadily (13.3%), and ear symptoms (tinnitus, hearing loss and aural fullness) (12.8%) were the most common concomitant symptoms in 211 hospitalized patients with dizziness as shown in Table 2.

**TABLE 2:** Concomitant symptoms in 211 hospitalized patients with dizziness

Concomitant symptoms	Number of patients	Percentage (%) (95% CI)
Nausea	102	48.3 (41.5–55.1)
Vomiting	72	34.1 (27.7–40.6)
Headache	28	13.3 (8.7–17.9)
Tinnitus, hearing loss, and aural fullness	27	12.8 (8.3–17.3)
Symptoms of nervous system defect	20	9.5 (5.5–13.5)
Walking unsteadily	28	13.3 (8.7–17.9)
Others	23	10.9 (6.7–15.1)
Nothing special	39	18.5 (13.2–23.8)

Abbreviation: CI, confidence interval.

### 3.3 Past medical history

The past medical history of 211 hospitalized patients with dizziness as their chief complaint is described in Table 3. Among them, 40.8% of patients had a past history of hypertension. Besides, diabetes (17.1%), cerebrovascular disease (stroke or transient ischemic attack) (16.6%), dyslipidemia (15.2%), and coronary heart disease (11.4%) were the most common diseases in past medical history of 211 hospitalized patients with dizziness. However, in 30.3% of patients, no significant past medical history was reported.

**TABLE 3:** Past medical history of 211 hospitalized patients with dizziness

Past medical history	Number (%)
Hypertension	86 (40.8)
Diabetes	36 (17.1)
Dyslipidemia	32 (15.2)
Anxiety and depression	5 (2.4)
Headache	6 (2.8)
Coronary heart disease	24 (11.4)
Stroke/transient ischemic attack	35 (16.6)
Ear disease	10 (4.7)
Head trauma	1 (0.5)
Others	21 (10.0)
Nothing special	64 (30.3)

### 3.4 Physical examination and auxiliary examination

Physical examination and auxiliary examination for 211 hospitalized patients with dizziness as the chief complaint are described in Table 4. In terms of physical examination, 29.4% of patients with dizziness as the chief complaint in our department of neurology had neurological defect symptoms. Dix‐Hallpike test was positive in 24.6% of patients. Romberg's sign was positive in 11.4% of patients. Directional nystagmus (2.4%), vision changes (1.4%), and hearing disorders (8.5%) were relatively rare symptoms. Unfortunately, there was no record of head impulse, nystagmus, test of skew (HINTS) test in all 211 patients. In terms of auxiliary examination, 60.2% of patients underwent a brain magnetic resonance imaging (MRI) examination. In addition, brain computed tomography (CT) (31.8%), carotid duplex ultrasound (30.8%), and echocardiography (28.0%) were common auxiliary examination methods in dizzy patients, whereas vestibular function tests (2.4%) and head‐up tilt test (1.4%) were rarely used.

**TABLE 4:** Physical examination and auxiliary examination of 211 hospitalized patients with dizziness as the chief complaint

Physical examination and auxiliary examination	Number of patients (%)
Nystagmus	5 (2.4)
Dix‐Hallpike	52 (24.6)
HINTS	0 (0)
Romberg's sign	24 (11.4)
Neurological defect symptoms	62 (29.4)
Vision changes	3 (1.4)
Hearing disorders	18 (8.5)
Negative physical examination	128 (60.7)
Brain CT	67 (31.8)
Brain MRI	127 (60.2)
Carotid duplex ultrasound	65 (30.8)
Echocardiography	59 (28.0)
Vestibular function tests	5 (2.4)
Head‐up tilt test	3 (1.4)

Abbreviations: CT, computed tomography; HINTS, head impulse, nystagmus, test of skew; MRI, magnetic resonance imaging.

### 3.5 Clinical diagnosis

Table 5 provided a description of clinical diagnosis of 211 hospitalized patients with dizziness as the chief complaint. Benign paroxysmal positional vertigo (24.2%) was the most common disease in 211 dizzy patients. Stroke/transient ischemic attack (19.0%) was the second leading cause of dizziness in our department of neurology. Other diseases such as vestibular migraine (2.8%), Meniere's disease (0.9%), persistent postural‐perceptual dizziness (0.9%), and sudden deafness with vertigo (0.5%) were quite rare in patients with dizziness as the chief complaint. However, up to 44.1% of patients with dizziness were undiagnosed.

**TABLE 5:** Clinical diagnosis of 211 hospitalized patients with dizziness as the chief complaint

Clinical diagnosis	Number of patients	Percentage (95% CI)
Benign paroxysmal positional vertigo	51	24.2 (18.3–30.0)
Stroke/transient ischemic attack	40	19.0 (13.6–24.3)
Vestibular migraine	6	2.8 (0.6–5.1)
Meniere's disease	2	0.9 (0.4–2.3)
Persistent postural‐perceptual dizziness	2	0.9 (0.4–2.3)
Sudden deafness with vertigo	1	0.5 (0.5–1.4)
Syncope	1	0.5 (0.5–1.4)
Postural hypotension	1	0.5 (0.5–1.4)
Others	14	6.6 (3.2–10.0)
Unable to specify	93	44.1 (37.3–50.8)

Abbreviation: CI, confidence interval.

### 3.6 Treatment effect

Treatment effect of 211 patients with dizziness as the chief complaint is described in Table 6. Among them, 97.2% of all 211 patients were in improved recovery. Two patients made full recovery, and three patients were discharged from hospital without any treatment. Only one patient died.

**TABLE 6:** Treatment effect of patients with dizziness (*n* = 211)

Treatment effect	Number (%)
Full recovery	2 (0.9)
Improved recovery	205 (97.2)
Discharged from hospital without treatment of dizziness	3 (1.4)
Deceased	1 (0.5)

## 4 DISCUSSION

As one of the most common problems encountered in department of neurology, dizziness is an ambiguous term that includes a wide array of medical disorders, so it is of great importance to have a good understanding of the characteristics of dizziness diseases from the aspects of demographic characteristics, characteristics of symptoms, past medical history, physical examination and auxiliary examination, clinical diagnosis, and treatment effect. A striking finding from this study was that the hospitalized patients with dizziness as the chief complaint made up a large proportion of all hospitalized patients in the department of neurology. Gender differences exist in the occurrence of dizziness, with a higher incidence in female than male patients. These findings were in accordance with the data from previous studies (Chang et al., 2018; Wojtczak et al., 2017). However, our study shows that there was no significant difference in the frequency of dizziness among different age groups between male cases and female cases.

According to the patient history, dizziness symptoms of these patients can be generally classified into four categories: vertigo, disequilibrium, pre‐syncope, or lightheadedness (Post & Dickerson, 2010). However, symptom types might substantially overlap in individual patients. Kerber et al. (2017) found that substantial overlap of dizziness types exists among US adults with dizziness. Usually, vertigo symptom will occur in bouts lasting for a short time. People who complained of vertigo symptom may also report more than one type of dizziness (Kerber et al., 2017). In our study, the primary dizziness type was used as the classification criterion. Vertigo and light‐headedness were more common dizziness types in our department of neurology. Disequilibrium was relatively less common. Pre‐syncope was the primary dizziness symptom reported by only three patients. Our findings suggest that the traditional classification based on dizziness types may lead to a difficult clinical diagnosis, since patients have difficulties describing the quality of their symptoms but they can more consistently identify the timing and triggers (Muncie et al., 2017).

Concomitant symptoms may indicate certain diseases and some of common dizziness are also diagnosed based on symptoms. Therefore, it is important to ask detailed questions about concomitant symptoms in dizzy patients. However, some concomitant symptoms are relatively specific for diagnosis of the cause of dizziness. Headache in dizzy patients may suggest a possible diagnosis of vestibular migraine (Lempert et al., 2022). Tinnitus, hearing loss, and aural fullness in dizzy patients may suggest Meniere's disease or other ear diseases (Basura et al., 2020). In our study, nausea and vomiting were the most common concomitant symptoms in dizzy patients, but these two symptoms were nonspecific and could be found in many other diseases. In contrast, specific concomitant symptoms (such as headache for vestibular migraine, symptoms of nervous system defect for cerebrovascular disease, tinnitus, hearing loss, and aural fullness for ear diseases) were common in dizzy patients.

Past medical history could help much in making a correct diagnosis for dizzy patients. Hypertension was the most common past history in hospitalized patients with dizziness in the department of neurology. Diabetes, dyslipidemia, coronary heart disease, and stroke/transient ischemic attack were also relatively common diseases in the past medical history of dizzy patients. On the contrary, headache, ear diseases, headache trauma, anxiety, and depression were less common. There are two possible reasons. First, dizzy patients with ear symptoms may have been admitted to the department of otology. Second, some patients with headache, head trauma, anxiety, and depression might be unwilling to be hospitalized for further diagnosis and treatment. This was different from otolaryngology and the emergency department (Ljunggren et al., 2018; Mandge et al., 2020; Yardley et al., 1998).

Some signs can often be found in physical examination and auxiliary examination in dizzy patients. Neurological defect symptoms were most common positive sign in dizzy patients and Dix‐Hallpike test was simple and important for diagnosis of benign paroxysmal positional vertigo. In our study, Dix‐Hallpike test was not used very often, only about one in five. Acute vestibular syndrome (AVS) is common in department of neurology, and physicians often use HINTS test to make a differential diagnosis of acute vestibular syndrome, especially to differentiate vestibular neuritis (the most common cause of an AVS) from ischemic stroke involving the cerebellum or the brainstem (the second most common cause of an AVS) (Kattah, 2018). Patients with stroke/transient ischemic attack often present with central HINTS signs (normal head‐impulse test, direction‐changing gaze‐evoked nystagmus, or pronounced skew deviation) (Kim et al., 2022). However, in our study, there was no record of using HINTS test, which can be explained by following several possible reasons. First, our neurologist did not know much about the HINTS test or could not perform the HINTS test. Then, physicians relied too heavily on imaging tests like CT and MRI. Third, physicians did not understand the specificity and sensitivity of HINTS test in diagnosis of stroke. Thus, almost one‐third of patients were performed with brain CT scan and almost two‐thirds of patients underwent brain MRI test. Carotid duplex ultrasound and echocardiography were also common auxiliary examination methods in dizzy patients.

Similar to a previous study, benign paroxysmal positional vertigo was also the most common disease among dizzy patients in our study (Patino et al., 2022), and stroke/transient ischemic attack was the second most common dizziness disorder. Only some patients were diagnosed with vestibular migraine, and fewer patients were diagnosed with Meniere's disease, postural‐perceptual dizziness, sudden deafness with vertigo, syncope, or postural hypotension. Our results were different from Hanley and Dowd's study in general practice (Hanley & Dowd, 2002). Unfortunately, a large proportion of patients were discharged without a definite diagnosis.

As in many previous studies, our study showed that almost all hospitalized patients with dizziness could get good outcome after treatment, which also illustrates that most dizziness diseases were benign. However, death still occurred in a very small number of dizzy patients. Based on this fact, we should rule out some malignant diseases (such as stroke) as soon as possible and help these patients get the right treatment in time.

This was a retrospective study based on past medical records, so the findings were fairly objective. However, our study inevitably has some limitations as well. First, our study was conducted among hospitalized patients in a department of neurology, so the results can only reflect the characteristics of hospitalized patients with dizziness in one department, was different from population‐based studies and other studies from otolaryngology department or emergency department. In addition, without a standardized design in advance, these medical records varied considerably in quality. Therefore, we plan to conduct a randomized controlled trial in the near future.

## 5 CONCLUSION

Dizziness was one of most common clinical symptoms. The hospitalized patients with dizziness as the chief complaint accounted for 11.5% of all hospitalized patients in the department of neurology. Dizziness was more common in women than in men. Vertigo and light‐headedness were the most common dizziness types in the department of neurology. Some concomitant symptoms (such as headache, ear symptoms) and past medical history were critical to the diagnosis of dizziness. Usually, some signs can be found in physical examination and auxiliary examination in dizzy patients. Therefore, it was necessary to master some simple bedside physical examinations (such as HINTS, Dix‐Hallpike) to differentiate dizziness diseases. Benign paroxysmal positional vertigo was the most common disease in dizzy patients in our study and stroke/transient ischemic attack was the second most common dizziness disorder. Although the prognosis of most dizzy patients was good, some malignant dizziness diseases also need to be excluded.

## CONFLICTS OF INTEREST

The authors declare no conflict of interest.

### PEER REVIEW

The peer review history for this article is available at https://publons.com/publon/10.1002/brb3.2864.

## Data Availability

All data used during the study are available from the corresponding author by request.
